# Determinants of under‐five pneumonia in randomly selected health facilities at Aleta Wondo Woreda, Sidama Region Ethiopia: Case–control study

**DOI:** 10.1111/crj.13725

**Published:** 2023-12-14

**Authors:** Tsegaye Alemu, Berihun Ayele, Mende Mensa Sorato

**Affiliations:** ^1^ School of Public Health Hawassa University Hawassa Ethiopia; ^2^ Pharma College Hawassa Ethiopia; ^3^ Department of Pharmacy, School of Medicine Komar University of Science and Technology Sulaimaniyah Iraq

**Keywords:** case–control study, children aged 2 to 59 months, determinants, pneumonia

## Abstract

**Introduction:**

Under‐five mortality reduction due to pneumonia is not Signiant, particularly in developing countries. Pneumonia contributed to 27.5% to 31.3% of health facility visits by children 2 to 59 months in Aleta Wondo Woreda. Previous studies have shown inconclusive evidence on determinants of pneumonia in children.

**Methods:**

An institution‐based unmatched case–control study was conducted to assess determinants of pneumonia among under‐five children at Aleta Wondo Woreda, Sidama Region.

**Result:**

One‐hundred forty‐five cases and 290 controls of children aged 2 to 59 months participated in the study. The mean ± (SD) age of the children was 18.81 months (2.1 ± 11.43) and 28.26 months (2.1 ± 16.007) for cases and controls, respectively. Only 56% (*n* = 145) of cases open house windows daily, whereas most 68.6% (*n* = 290) of controls house windows open daily. Ninety five (62.8%) of cases and 68.6% of controls were exclusively breastfed for 6 months. Household income ≥1500 Ethiopian birr (AOR = 0.45, 95% CI, 0.017–0.120, *p* < 0.000), child location outside of cooking house during cooking (AOR = 0.101, 95% CI, 0.43–0.238, *p* < 0.000), no formal education of the mother (AOR = 2.398, 95% CI, 1.082–5.316, *p* < 0.031), and presence of history of upper respiratory tract infections (URTIs) in last 2 weeks (AOR = 2.183, 95% CI, 1.684–5.273, *P* < 0.049) were determinants of pneumonia.

**Conclusion:**

Determinants of pneumonia in under‐five children were multifactorial (socioeconomic, nutritional, and environmental). Addressing these factors by involving all relevant stakeholders is important to reduce pneumonia‐related morbidity and mortality among under‐five children in the study area.

## Graphical abstract

Under‐five mortality reduction due to pneumonia is not Signiant, particularly in developing countries. Pneumonia contributed to 27.5 to 31.3% of health facility visits by children two to 59 months in Aleta Wondo Woreda. Determinants of pneumonia in under‐five children were multifactorial (socioeconomic, nutritional, and environmental).

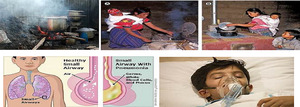

**Abbreviations**
CAP: community‐acquired pneumonia
CEO: chief executive officer
CHD: chronic heart disease
CI: confidence interval
COR: crude odd ratio
EFMOH: Federal Ministry of Health of Ethiopia
EOPD: Emergency Out Patient Department
GAPPD: Integrated Global Action Plan for Pneumonia and Diarrhea
HIV: human immune deficiency virus
HMIS: Health Management Information System
IMNCI: Integrated Management of Newborn and Childhood Illness
MUAC: mid‐upper arm in circumferences
OPD: Out Patient Department
SNNPR: Southern Nation Nationality Peoples Region
SSPS: statistical package for social sciences
UNICEF: United Nations International Children's Emergency Fund
URTI: upper respiratory tract infection
USA: United States of America
WHO: World Health Organization

## 1 INTRODUCTION

Pneumonia accounted for 16% of under‐five mortality (i.e., killed about 900 000 children in 2016); around 2200 child dies every day.^1^ Globally, 1 out of 71 children develop pneumonia every year.^2^ Every year, about 7 million under‐five children are admitted to hospitals with pneumonia, requiring urgent oxygen treatment to survive in low‐ and middle‐income countries.^3^ Most of the pneumonia‐related deaths were in developing countries due to limited access to evidence‐based interventions.^4^

Under‐five mortality reduction due to pneumonia is not Signiant, particularly in developing countries. Only a 54% decline in death was reported in the last two decades, while deaths due to diarrhea have shown a 64% decline.^2^, ^5^ Some studies showed increased hospital admissions globally and a rapid increase reported by WHO South‐East Asia. Ethiopia is one of five countries that accounted for 49% of global pneumonia deaths in 2015 (India, Nigeria, Pakistan, the Democratic Republic of the Congo, and Ethiopia).^6^

In Ethiopia, pneumonia contributes to 16.4% of all deaths of children under 5 years of age more than diarrhea, malaria, AIDS, and measles combined.^7^, ^8^ The country incorporated the pneumococcal conjugate vaccine into the expanded program on immunization in 2011 to prevent or reduce morbidity and mortality associated with childhood pneumonia.^9^

Previous studies indicated comorbid conditions, lack of exclusive breastfeeding, indoor air pollution, parental cigarette smoking, malnutrition, using charcoal for cooking, advanced maternal age, previous upper respiratory tract infections, poor socioeconomic status, keeping domestic animals inside the main house, lack of zinc supplementation, absence of a separate kitchen, father's education, history of diarrhea in child, household history of acute lower respiratory infection, and child born in rural areas as most common determinants for pneumonia in children.^2^, ^5^, ^10^, ^11^, ^12^, ^13^, ^14^, ^15^, ^16^

In Ethiopia, even interventions for under‐five pneumonia like immunization, proper nutrition, exclusive breastfeeding, zinc and vitamin A supplementation, appropriate complimentary feeding, safe drinking water, and good sanitation and control of environmental factors are undertaken by using health extension workers. But the available data show still under‐five pneumonia is the leading problem of economic, social, and economic burden those developing nations like Ethiopia.

Despite the continued effort to reduce the problem, pneumonia remains a challenge to the healthcare system of Ethiopia. The increasing burden of pneumonia in under‐five children in Ethiopia calls for an enduring solution to reduce and or prevent the problem. In addition to this, pneumonia in children contributes to a substantial economic burden on the affected society.^17^, ^18^ There is inadequate data on the determinants of pneumonia in Aleta Wondo Woreda. Identifying contributing factors is important to inform program managers, policymakers, and the general population. Therefore, this study aimed to identify the determinants of pneumonia among 2 to 59 months old children at a randomly selected health facility in Aleta Wondo Woreda, Sidama Region, Southern Ethiopia.

## 2 METHODS AND MATERIALS

### 2.1 Study design, area, and period

A facility‐based unmatched case–control study design was conducted from June 1 to 30, 2022, in the Aleta wondo woreda, which is located in the Sidama Region, Southern Ethiopia. The Aleta wondo woreda was found in southern Addis Ababa 336.0 km and 62.0 km from Hawassa as mentioned on citymeter.net. It is one of woreda in southern Ethiopia in the Sidama Region; it is bordered on the south by Daara woreda, on the west by Chuko, on the north by Dale and Wonsho, on east by Bursa, on the southeast by Hula woreda. Based on 2007 EC population projection of central statistical agency, a total population of the woreda was 188 976 of whom 96 640 are men followed by women 92 336. A total under‐five population in the woreda was 37 636.^15^ In the Aleta town administration, there is one public primary hospital and seven health centers on the rural woredas administration as data found from Sidama Region Health Bureau.

### 2.2 Population

#### 2.2.1 Source population

The source of cases and controls population consisted of children 2 months to 5 years of age living for a minimum of 6 months in Aleta wondo woredas kebeles Sidama Region, Southern Ethiopia.

#### 2.2.2 Study population

The *cases* were all under‐five children with diagnosed pneumonia by a determined respective physician or healthcare professional based on the Federal Democratic Republic of Ethiopia Ministry of Health Integrated Management of Newborn and Childhood Illness (IMNCI) guideline (adopted from WHO), who came for treatment service during the data collection period.^19^ Typical pneumonia is an acute infection of the lungs with symptoms of coughing, fast breathing, and chest indrawing defined by the World Health Organization (WHO) Integrated Management of Childhood Illness (IMNC).^19^ Pneumonia is all under‐five cases visit the health facility with cough or difficulty of breathing and age‐specific fast breathing or consolidation or infiltration that are found on chest X‐ray,^19^ while c*ontrols* were children aged 2 to 59 months who presented for immunization, growth monitoring service, and visit for care other than pneumonia case.

### 2.3 Sample size determination

The sample size was calculated by using Epi Info version 7 (STATCALC program) and calculated the minimum number of cases and controls required by taking assumptions level of significance using 0.05, the power of the test using (1 − β) = 80%. The proportion of exposure among controls (p1) = 42.8% with the proportion of exposure among cases (p2) = 57.3% and AOR = 1.84 was inserted into the Epi Info formula to determine the sample size.^20^ Total sample size after a nonresponse rate of 10% consideration accordingly the final sample size was 440 (147 cases and 293 controls) by taking a 1:2 ratio of cases to controls.

### 2.4 Variables of the study

#### 2.4.1 Dependent variables

Dependent variables are determinants of pneumonia in under‐five children.

#### 2.4.2 Independent variables

Socioeconomic status‐related variables include living in a crowded house, parental cigarette smoking, keeping domestic animals inside the main house, using charcoal for cooking, extended family size, absence of a separate kitchen and or window in the kitchen, advanced maternal age, and father's education. Child‐related variables include comorbid conditions, lack of exclusive breastfeeding, malnutrition, previous URTIs, lack of zinc supplementation, age of the child, previous history of diarrhea, and household history of acute lower respiratory infection, whereas environment‐related variables include indoor air pollution and a house near the street.

### 2.5 Sampling technique

A clustered systematic random sampling technique involved health facilities in the Woreda including the town administration into a Hospital and the Health Center, the Aleta Wondo Town Primary Hospital selected, and the two health centers randomly taken by the lottery method (Wicho Health Center and Gowadamo Health Center) based on the number of clients/patients who visit each health facility during the previous 6 months from the health facility HMIS data. Then, the total sample size proportionally allocates to each health facility. Selected health facility data were collected by the well‐structured questionnaire attending out‐patient service under‐five children from a select health facility in Aleta Wondo woreda during the study period June 1 to 30, 2022. From the HMIS of each health facility, last 6 months total of 2502, 486, and 144 under‐five children visited Aleta Wondo Primary Hospital, Wicho Health Center, and Gowadamo Health Center, respectively, for service. Out of 2502 total cases attended Aleta Wondo Primary Hospital 689 (27.5%) were pneumonia cases. Out of 486 under‐five children visits to Wicho Health Center, 144 (29.6%) were pneumonia cases. Similarly from a total of 288 under‐five children visits to Gowadamo Health Center, 90 (31.3%) were pneumonia cases. From a total of three health facilities pneumonia cases adding get 923 taking a proportional sample from each health facility, from Aleta Wondo Primary Hospital (108 cases and 218 controls), Wicho Health Center (24 cases and 47 controls), and Gowadamo Health Center (15 cases and 28 controls) were included.^21^

### 2.6 Data collection procedure and instrument

The data were collected by using direct Interviewers to administer well‐structured questionnaires from sample study unit mothers or caregivers who visited under‐five children patients departments. The questionnaire was prepared by adapting from various literature reviews and related case–control studies done in northeast Ethiopia.^12^ The questionnaire was developed in English language and then translated into Sidamic language version and back to English to check for consistency. The Sidamic version was used to collect the data by face‐to‐face interview with the mother/caretaker to collect data on socio‐demographic and independent variables. Anthropometric measurements, the child's height (to the nearest 0.1 cm) and weight (to the nearest 0.1 g), were taken. The interview was carried out in a private room, which was prepared near the under‐five outpatient department. The nutritional status of the children was determined using enhanced nutritional action (ENA) software.^22^ The WHO (2006) growth standard to report principal anthropometric results and the global acute malnutrition standard was used to categorize a child's condition as stunted, wasted, or underweight.^23^

### 2.7 Data quality assurance and analysis

#### 2.7.1 Data quality assurance

The pretest was conducted by taking 10% of the total sample size and applying the questionnaire, and amendments were made based on findings. Three nurses received 1 day of training on data collection procedures and rules. Ongoing supervision was made by principal investigators during the data collection period. Language experts translated the questionnaire from English to the Sidamic version.

#### 2.7.2 Data analysis

All data were checked, coded, and entered into Epi‐data Version 6.2 and then exported to SPSS Version 25 for further analysis. The principal investigator checked the extent of outliers, the different statistical assumptions, and the appropriate correction mechanisms before analysis. The association of each independent variable was assessed with binary logistic regression, and the strength of their association was computed by an unadjusted odds ratio (COR, 95% CI). Variables showing statistically significant associations with the outcome variables (up to p = 0.2) were considered as potential determinants of pneumonia and simultaneously subjected to stepwise multiple logistic regression models to determine the significant independent determinants of pneumonia. A p‐value < 0.05 was considered statistically significant.

## 3 RESULT

### 3.1 Sociodemographic characteristics

One‐hundred forty‐five cases and 290 controls of children aged 2 to 59 months participated in the study, making the response rate 98.6% and 99% for cases and controls, respectively. The mean ± (SD) age of the child was 18.81 months (2.1 ± 11.43) and 28.26 months (2.1 ± 16.007) for cases and controls, respectively. The gender of the children was 51.7% (*n* = 145) of cases and 49.3% (*n* = 290) of controls were male. The mean ± (SD) age of mothers was 26.66 years (2.1 ± 6.24) and 25.64 years (2.1 ± 4.68) for cases and controls, respectively. The educational status of mothers 7% of cases and 5.5% of controls were illiterate (Table 1).

**TABLE 1:** Sociodemographic characteristics of under‐five children, in selected health facilities at Aleta Wondo Woreda, Sidama Region, Ethiopia 2022 (*N* = 145 cases and 290 controls).

Variables		Cases (%)	Controls (%)
Age of child in months	Less than 12 months	55 (37.9)	122 (41.7)
	12–60 months	90 (62.1)	168 (57.3)
Sex of child	Male	75 (51.7)	143 (49.3)
	Female	70 (48.3)	147 (50.7)
Age of the mother	15–24	53 (36.6)	111 (38.3)
	25–34	79 (54.4)	03 (35.5)
	Greater than 34	13 (9)	76 (26.2)
Education status of mother education	No formal education attended	10 (7)	16 (5.5)
	Primary (1–4 compete)	21 (14.5)	40 (13.8)
	Primary (5–8 completed)	38 (26.2)	59 (20.3)
	Secondary (9–12 completed)	65 (44.8)	107 (36.9)
	Higher education	11 (7.8)	68 (23.5)
Mother's occupation	Housewife	64 (44)	147 (50.8)
	Government employee	17 (11.7)	23 (7.9)
	Merchant	11 (7.6)	34 (11.7)
	Others (farmers, daily workers, and students)	53 (36.7)	86 (29.6)
Father's occupation	Government employee	23 (15.8)	36 (12.4)
	Merchant	34 (23.6)	94 (32.4)
	Farmer	55 (37.9)	108 (37.2)
	Others (drivers, students, private, and factory workers)	33 (22.7)	52 (17.9)
Family size	<4	31 (21.3)	112 (38.6)
	≥4	114 (78.7)	178 (61.4)
Residence	Rural	87 (60)	192 (66.2)
	Urban	58 (40)	98 (33.8)
Household monthly income	<1500	70 (48.3)	129 (44.5)
	≥1500	75 (51.7)	161 (55.5)

### 3.2 House and environment‐related characteristics

**FIGURE 1:**
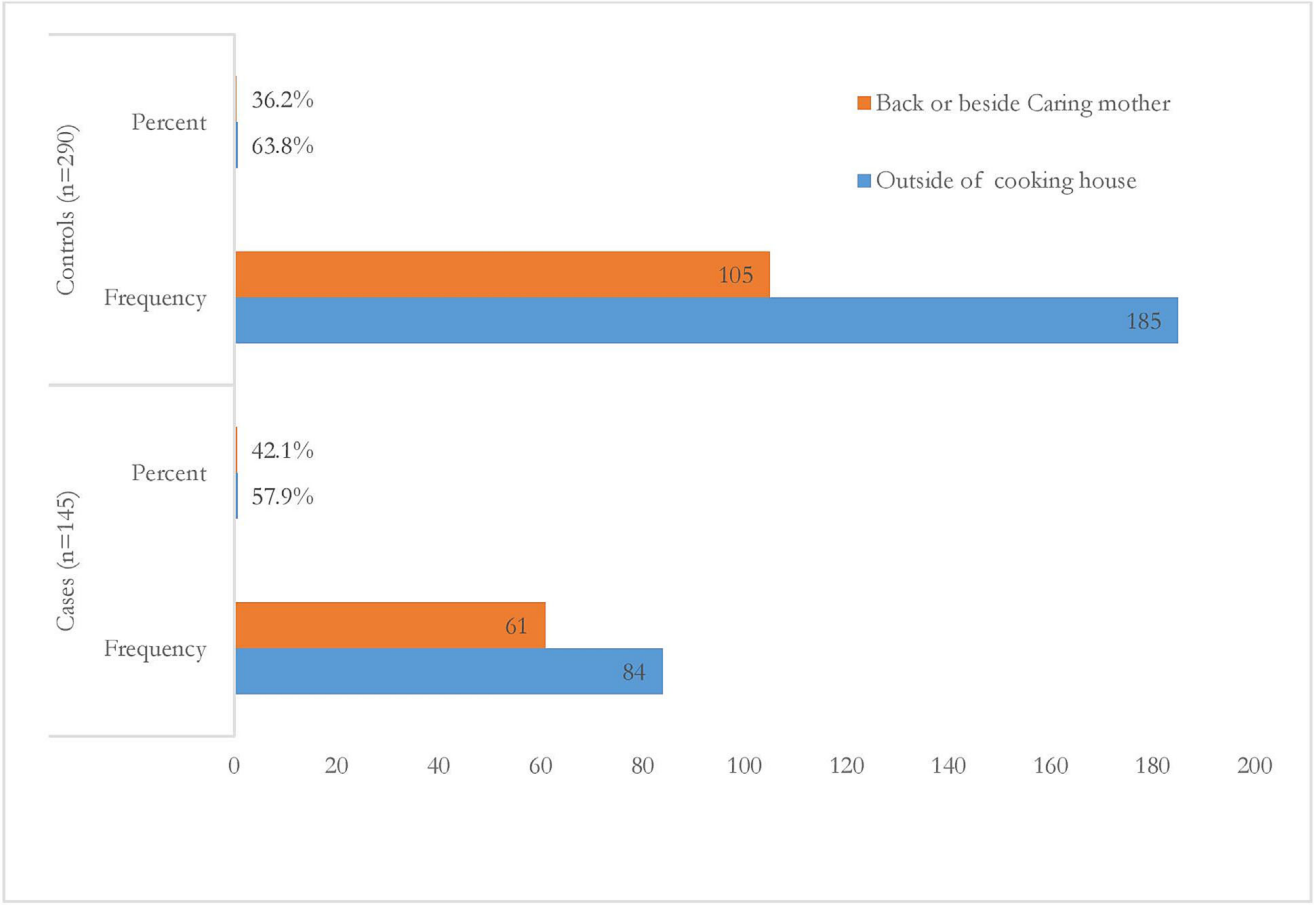
Child location during cooking among respondents in selected health facilities at Aleta Wondo Woreda, Sidama region, Ethiopia, 2022 (*N* = 145 cases and *N* = 290 controls).

Only 56% (*n* = 145) of cases open house windows daily, whereas most 68.6% (*n* = 290) of controls house windows open daily. The family cooks food in the living room in 60% of cases and only in 33.3% of controls. Most respondents have used it as a fuel source for cooking wood/crops about 92.2% of cases and 60% of controls. A mother caring back or besides during food cooking in 42.1% of cases and about 36.2% of controls (Table 2 and Figure 1).

**TABLE 2:** Housing and environmental‐related characteristics of respondents in selected health facilities at Aleta Wondo Woreda, Sidama region, Ethiopia, 2022 (*N* = 145 cases and *N* = 290 controls).

Variables			Cases (%)	Controls (%)
Place of cooking		Living room	87 (60)	96 (33.3)
		Kitchen	58 (40)	194 (66.7)
Windows in house		Less than 2 windows in the house	47 (32.4%)	147 (50.6)
		2 and more windows in the house	98 (67.5%)	143 (49.3)
Fuel used for cooking	Charcoal	Yes	57 (39.3)	123 (42.4)
		No	88 (60)	167 (57.6)
	Wood/crop	Yes	134 (92.4)	174 (60)
		No	11 (7.6)	116 (40)
	Electricity	Yes	57 (39.3)	94 (32.4)
		No	88 (60.7)	196 (67.6)
Family history of smoking cigarette		Yes	7 (4.8)	18 (6.2)
		No	138 (95.2)	272 (93.8)
The house window open		Daily	80 (56)	199 (68.6)
		Usual	63 (44)	91 (31.4)
Place of child sleep		Separate room	79 (54.5)	167 (57.6)
		Same room for food cooking	66 (45.5)	123 (42.4)
Child location during cooking		Outside of the cooking house	84 (57.9)	185 (63.8)
		Back or beside caring mother	61 (42.1)	105 (36.2)
Kind of toilet		Pit latrine	114 (78.6)	246 (84.8)
		Open field	25 (17.3)	31 (10.7)
		Ventilate improved pit latrine	6 (4.1)	13 (4.5)

### 3.3 The child and parent‐related characteristics

**FIGURE 2:**
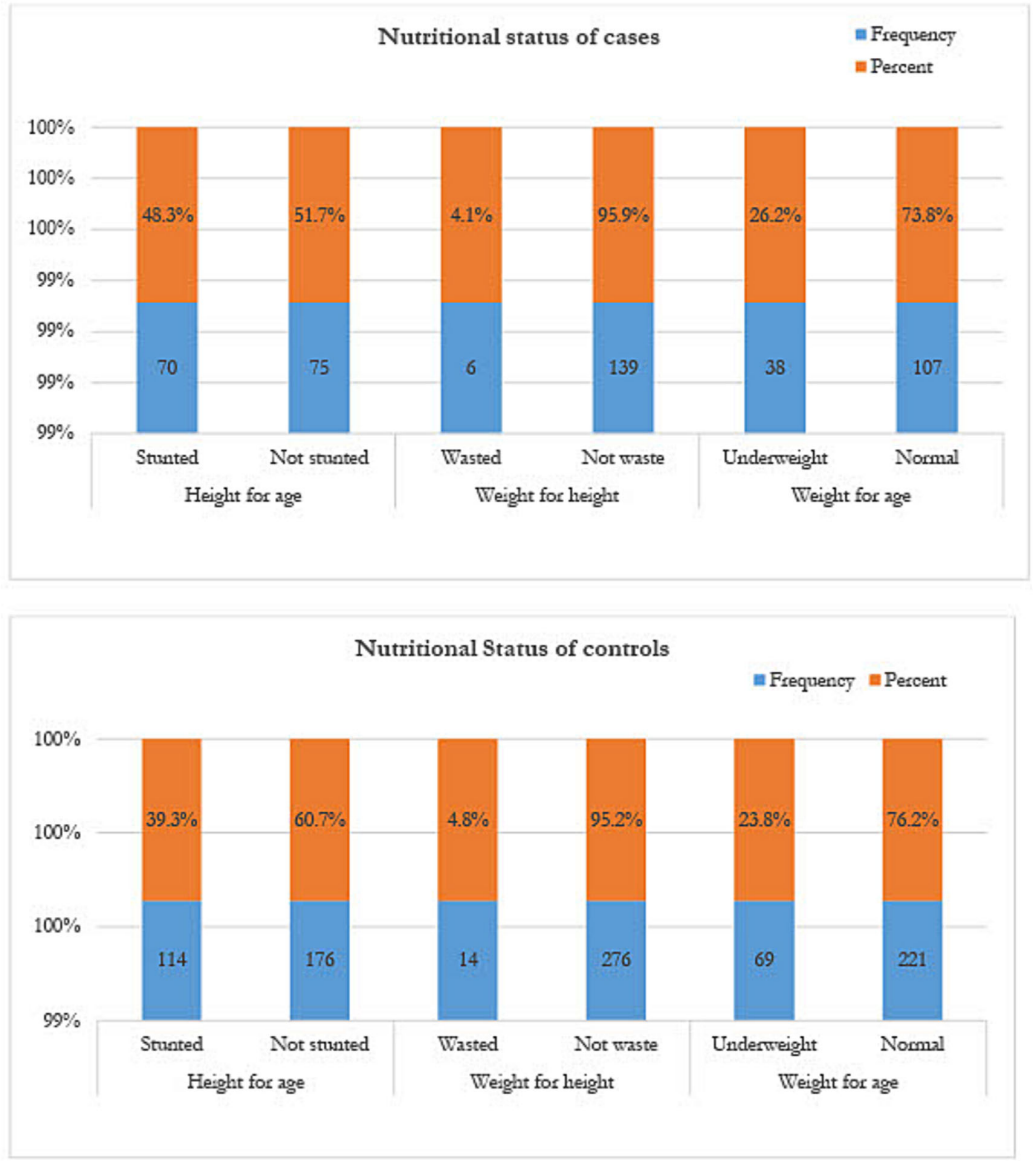
Nutritional status of under‐five children in selected health facilities at Aleta Wondo Woreda, Sidama Region, Ethiopia, 2022 (*N* = 145 cases and *N* = 290 controls).

Of the nutritional status of under‐five children, 48.3% (*n* = 145) of cases and 39.3% (*n* = 290) of controls were stunted; 26.2% of cases and 23.8% of controls were underweight; and 4.2% of cases and 14 four point eight percent of controls were wasted (too thin for his or her height) (Figure 2).

About 95 children 62.8% of cases and 68.6% of controls were exclusively breastfed for 6 months. Sixty children 41.4% of cases and 128 (44.1%) controls had a history of diarrhea in the last 2 weeks. About 38 (26.2%) of cases and 26.9% of controls had upper respiratory tract infections (URTIs) within 2 weeks before the facility visit. The family pneumonia attack experienced the 2 weeks before visiting a health facility were 50, 34.5% of cases and 29.6% controls (Table 3).

**TABLE 3:** Children and parental‐related characteristics of respondents in selected health facilities at Aleta Wondo Woreda, Sidama Region, Ethiopia, 2022 (*N* = 145 cases and *N* = 290 controls).

Variable		Cases (%)	Controls (%)
Breastfeed first 6 months	Exclusive BF	91 (62.8)	199 (68.6)
	Mixed BF	54 (37.2)	91 (31.4)
History of pneumonia in family within 2 weeks	Yes	50 (34.5)	86 (29.6)
	No	95 (65.5)	204 (70.4)
Diarrhea within last 2 weeks	Yes	60 (41.4)	128 (44.1)
	No	85 (58.6)	162 (55.9)
Illness of URTIs within 2 weeks	Yes	38 (26.2)	78 (26.9)
	No	107 (73.8)	212 (73.1)
Vaccination status of the child	Fully vaccinated	94 (64.8)	183 (63.1)
	Up to date	37 (25.5)	77 (26.6)
	Partially vaccinated	13 (8.9)	28 (9.7)
	Unvaccinated	1 (0.8)	2 (0.6)

### 3.4 Mean difference of exposure between cases and controls

We conducted an independent sample *t* test to evaluate the equality of variance and mean difference among cases and controls to exposure variables. To check the similarity of variance among cases and controls, Levene's test for equality of variances was used (i.e., equal variances, *p* > 0.05, and unequal variances *p* < 0.05). Similarly, to identify the mean difference among cases and controls *t* test for equality of means was used (i.e. *p* < 0.05 means a significant difference in the means of the two sample populations tested (cases and controls). Since the variance is greater than 4 in almost all of the test variables, we assumed unequal variance and used a one‐sample *t* test.^24^ Exposures associated with increased risk of pneumonia in under‐five children were maternal education (*p* < 0.001), age of the child in months (*p* < 0.006), income of family (*p* < 0.000), place of cooking (*p* < 0.006), separation of kitchen (*p* < 0.000), presence of windows in the house (*p* < 0.000), MUAC (*p* < 0.012), and history of lower respiratory tract infections (*p* < 0.007) (Table 4).

**TABLE 4:** Independent sample *t* test of respondents in selected health facilities at Aleta Wondo Woreda, Sidama Region, Ethiopia, 2022 (*N* = 145 cases and *N* = 290 controls).

Independent samples test		Levene's test for equality of variances		T‐test for equality of means						
		F	Sig.	T	Df	Sig. (2‐tailed)	Mean difference	Std. error difference	95% CI of the difference	
									Lower	Upper
Maternal education	EVA	14.294	0.000	−3.129	433	0.002	−0.24483	0.07824	−0.39861	−0.09104
	EVNA			−3.298	331.644	**0.001** *	−0.24483	0.07424	−0.39087	−0.09878
Family residence	EVA	7.249	0.007	1.491	433	0.137	0.072	0.049	−0.023	0.168
	EVNA			1.469	276.961	0.143	0.072	0.049	−0.025	0.169
Age of child in month	EVA	25.383	0.000	−2.854	433	0.005	−0.13103	0.04592	−0.22129	−0.04078
	EVNA			−2.744	260.011	**0.006** *	−0.13103	0.04776	−0.22507	−0.03699
Income of family	EVA	120.096	0.000	4.894	433	0.00	0.234	0.048	0.140	0.329
	EVNA			5.213	341.177	**0.000** *	0.234	0.045	0.146	0.323
Place of food cooking	EVA	17.050	0.000	−2.817	433	0.005	−0.138	0.049	−0.234	−0.042
	EVNA			−2.761	273.156	**0.006** *	−0.138	0.050	−0.236	−0.040
Child location during cooking	EVA	4.536	0.034	−1.186	433	0.236	−0.059	0.049	−0.156	0.039
	EVNA			−1.174	280.903	0.241	−0.059	0.050	−0.157	0.040
Separation of kitchen from main house	EVA	51.024	0.000	−4.309	433	0.000	−0.214	0.050	−0.311	−0.116
	EVNA			−4.427	309.917	**0.000** *	−0.214	0.048	−0.309	−0.119
Presence of windows in the house	EVA	40.485	0.000	3.662	433	0.000*	0.183	0.050	0.085	0.281
	EVNA			3.741	305.132	**0.000**	0.183	0.049	0.087	0.279
MUAC of children	EVA	4.135	0.043	2.665	433	0.008	0.45221	0.16970	0.11867	0.78574
	EVNA			2.539	254.117	**0.012** *	0.45221	0.17809	0.10149	0.80292
History of ARTI	EVA	30.801	0.000	−2.895	433	0.004	−0.114	0.039	−0.191	−0.037
	EVNA			−2.697	240.282	**0.007** *	−0.114	0.042	−0.197	−0.031

*Note*: Bold numbers are statistically significant.

Abbreviations: EVA, equal variance assumed; EVNA, equal variance not assumed; LRTI, acute respiratory tract infection; MUAC, mid upper arm circumference.

* The variable is significantly different between cases and controls.

### 3.5 Determinants of pneumonia in under‐five children

In bivariate analysis, place of residence (COR = 2.654, 95% CI, 1.145–5.548), household income ≥1500 Ethiopian birr (COR = 1.639, 95% CI, 1.001–3.759), child location during cooking (COR = 0.560, 95% CI, 0.373–0.842), no formal education of the mother (COR = 2.270, 95% CI, 1.284–8.568), family history of smoking (COR = 2.476, 95% CI, 1.587–5.786), presence of diarrhea in last 12 weeks (COR = 6.564, 95% CI, 3.975–10.865), and history of URTI in the family in last 2 weeks (COR = 3.354, 95% CI, 2.543–5.764) were associated with pneumonia. In multivariable logistic regression, household income ≥1500 Ethiopian birr (AOR = 0.45, 95% CI, 0.017–0.120, *p* < 0.000) when compared with monthly income below 1500 Ethiopian birr, child location outside of cooking house during cooking (AOR = 0.101, 95% CI, 0.43–0.238, *p* < 0.000) when compared to carrying in back or putting besides caring mother were protective factors against pneumonia, while no formal education of the mother (AOR = 2.398, 95% CI, 1.082–5.316, *p* < 0.031) when compared to college and above and a history of URTI in the family in the last 2 weeks (AOR = 2.183, 95% CI, 1.684–5.273, *P* < 0.049) when compared to no history of URTI are risk factors for pneumonia (Table 5).

**TABLE 5:** Bivariate and multivariable logistic regression model for determinants of children pneumonia among age 2–59 months of age in selected health facilities at Aleta Wondo Woreda Sidama Region, Ethiopia, 2022.

Variables		Cases, *n* (%)	Controls, *n* (%)	COR (95% CI)	AOR	95% CI	*P* value
Residence	Rural	87	192	2.654 (1.145–5.548)*	0.695	0.438–1.102	0.122
	Urban	58	98	1	1		
Household monthly income	<1500	70	129	1	1		
	≥1500	75	161	1.639 (1.001–3.759)*	0.045	0.017–0.120	**0.000** *
Child location during cooking	Outside of the cooking house	84 (57.9)	185 (63.8)	0.560 (0.373–0.842)*	0.101	0.043–0.238	**0.000** *
	Back or beside caring mother	61 (42.1)	105 (36.2)	1			
Education status of the mother	No formal education	10	16	2.270 (1.284–8.568)*	2.398	1.082–5.316	**0.031** *
	Primary school completed	21	40	1.364 (0.684–4.762)*	2.799	0.885–8.851	0.080
	Secondary	65	107	1.263 (0.124–9.465)*	1.462	0.617–3.464	0.388
	College and above	11	68	1	1		
Family history of smoking cigarette	Yes	7	18	2.476 (1.587–5.786)	0.542	0.197–1.488	0.234
	No	138	272	1	1		
Diarrhea within the last 2 weeks	Yes	60	128	6.564 (3.975–10.865)*	1.092	0.681–1.753	0.714
	No	85	162	1	1		
History of family ARTI within 2 weeks	Yes	38	78	3.354 (2.543–5.764)*	2.183	1.684–5.273	**0.049**
	No	107	212	1	1		

*Note*: Bold numbers are statistically significant.

Abbreviations: AOR, adjusted odds ratio; COR, crude odds ratio.

* Statistically significant at *p* < 0.05.

## 4 DISCUSSION

This unmatched case–control aimed to identify determinants of community‐acquired pneumonia among under‐five children in the selected health facilities at Aleta Wondo Woreda Sidama region southern Ethiopia. Based on our primary assessment, the proportion of pneumonia‐related health facility visits in the last 6 months during the study period was 923 (29.467% ± 1.9, SD). This is in line with a systematic review and meta‐analysis conducted among under‐five children in East Africa involving 34 studies that showed the pooled prevalence of pneumonia in East Africa was 34%.^5^ However, the finding is higher than studies conducted among 560 systematically selected under‐five children in Dessie City, 17.1%,^8^ and prevalence and associated risk factors of pneumonia in under‐five children in Gondar Referral Hospital, 18.5%.^25^ The difference could be explained by the geographical variation of study areas. A spatial and multilevel analysis of common childhood illnesses and their associated factors among under‐five children in Ethiopia showed significant variation among geographical regions with common childhood illnesses.^26^

### 4.1 Determinants of pneumonia in under‐five children

Children from families with household income ≥ 1500 Ethiopian birr 54% less (AOR = 0.45, 95% CI, 0.017–0.120, *p* < 0.000) are less likely to develop pneumonia when compared with families with monthly income below 1500 Ethiopian birr. This is supported by evidence from a case–control study conducted at Adama Hospital Medical College that showed children from households with low monthly incomes were more likely to develop pneumonia.^27^ Another study among under‐five children in Ethiopia also showed that poverty is significantly associated with common childhood illnesses.^26^

Child location outside of the cooking house during cooking is a protective factor against pneumonia. Children placed outside, or in separate rooms during cooking were 90% less likely (AOR = 0.101, 95% CI, 0.43–0.238, *p* < 0.000) to develop pneumonia when compared to children carried in the back of their mother or placed beside a caring mother. This is supported by evidence from other studies, children sleeping in place same room as cooking food were experiencing pneumonia more likely than those children sleeping in a separate room,^20^ a systematic review and meta‐analysis conducted among under‐five children in East Africa involving 34 studies showed that use of wood as a fuel source, cook food in the living room, caring of a child on mother during cooking,^5^ another cross‐sectional study conducted in Northwest Ethiopia showed that the prevalence of childhood lower acute respiratory infection lower among children living in homes with chimney, eaves space, and improved cook‐stove and increased with cow dung fuel use, and child spending time near stove during cooking.^28^ An unmatched case–control study conducted in Worabe town showed that the absence of a chimney in the cooking room was positively associated with pneumonia.^29^ Another case–control study conducted in Wolaita‐Sodo Ethiopia showed that unclean fuel users for cooking, poorly ventilated houses, and carrying of a child while cooking were risk factors of acute respiratory infection in under‐five children.^30^ This can be explained by the exposure to indoor air pollution that makes the children susceptible to pneumonia attack, so the study result shows that children from families sleeping in the same cooking room of food may increase the risk of developing the attack of pneumonia.

Maternal education is protective against under‐five pneumonia; children of mothers with no formal education were 2.4 times (AOR = 2.398, 95% CI, 1.082–5.316, *p* < 0.031) more likely to develop pneumonia than children with educated mothers who attended college and above. This is in line with evidence from a similar study conducted at Gondar University Hospital that showed an increased odds of pneumonia was associated with poor maternal education.^20^ In addition to this, maternal education could affect other risk factors for pneumonia, like the nutritional status, smoking status of the mothers, and economic status of households.^12^, ^31^, ^32^ Findings from the 2016 Ethiopian demographic health survey also showed that mothers' secondary school education is associated with a lower risk of under‐five acute respiratory infections.^33^ Another study also revealed result under‐five children's pneumonia attacks are more likely related to those illiterate mothers than educated mothers.^34^ Therefore, empowering mothers through education in the study area will reduce the burden of pneumonia in under five children.

In addition to this, children having a family member with a history of URTIs in the last 2 weeks was 2.1 times more (AOR = 2.183, 95% CI, 1.684–5.273, *P* < 0.049) to develop pneumonia when compared to no history of URTIs. This result is supported by a study from Urban Areas of the Oromia Zone, Amhara Region, and the WHO.^2^ A case–control study conducted among under‐five children at Debre Markos referral hospital showed that having a family member with URTI in the last 2 weeks was a risk for pneumonia.^35^ Another population‐based analysis among 475 000 Children from 30 Low‐ and Middle‐Income Countries showed that using wood for cooking is associated with increased odds of URTI when compared to using charcoal.^36^ This further strengthens the association between pneumonia in under‐five and indoor air pollution.

### 4.2 Limitations of the study

The findings of this study should be applied in light of its limitations. The small observation in certain categories may reduce the precision of the study. The second limitation is being an institution‐based case–control study that can limit generalizability.

## 5 CONCLUSION

The burden of pneumonia among under‐five children in the study area was high (pneumonia accounted for one‐third of health facility visits). Household income ≥1500 Ethiopian birr, child location outside of the cooking house during cooking were protective factors against childhood pneumonia, whereas no formal education of the mother and the presence of a history of URTI in the family in the last 2 weeks were risk factors associated with pneumonia in under five children. Therefore, all health institutions should promote early treatments and prevention of acute lower respiratory infections of children in the health facility and at the household level. Providing health education about household cooking conditions and empowering mothers economically and educationally is important to reduce the current burden of pneumonia in under five children. In addition to this, researchers should conduct multicenter studies with a better methodology to elucidate the causal link between pneumonia and socioeconomic and environmental factors.

## AUTHOR CONTRIBUTIONS

Tsegaye Alemu, Berihun Ayele, and Mende Mensa Sorato contributed in the conception, study design, data collection at the hospital, data analysis, and interpretation. Tsegaye Alemu and Mende Mensa Sorato edited the manuscript and critically reviewed the manuscript. All authors read and approved the manuscript before submission.

## CONFLICT OF INTEREST STATEMENT

The authors declare no conflict of interest.

## ETHICS STATEMENT

Ethical clearance was obtained from the Institutional Review Board of the Pharma College from the public health department post‐graduate program. A formal letter of cooperation was written to Sidama Region Health Bureau. All authors read the full version of this manuscript and agreed to publish.

## Data Availability

All the data reported in the manuscript are publicly available at the official request of the principal investigator upon acceptance of the manuscript.
